# Losing the thread: experiences of cognitive decline in Alzheimer's disease

**DOI:** 10.1192/bjp.2022.184

**Published:** 2023-04

**Authors:** Paul Bebbington

**Affiliations:** Emeritus Professor, University College London Division of Psychiatry, UK

**Keywords:** Dementia, social functioning, stigma and discrimination, phenomenology, organic syndromes

## Abstract

In an initiative to reduce stigma, an academic psychiatrist comes out of the dementia closet: describing his own experience of developing Alzheimer's disease, the accompanying memory problems, the restriction of some of his activities, emotional lability and his increasing reliance on others.

**Lived experience narratives of dementia**

I am saddened to bring this editorial to the readership. It is personal, about a colleague and friend. Professor Paul Bebbington, an eminent social and epidemiological psychiatrist, shows courage in talking about his diagnosis, bringing personal experiences to challenge stigma and secrecy associated with the diagnosis. According to the World Health Organization (WHO), more than 55 million people worldwide live with dementia, the seventh leading cause of death. Alzheimer's disease accounts for 60–80% of dementia and can affect people under the age of 65 years, although the incidence increases with age. As we are living longer, so the prevalence of dementia is increasing. WHO estimates 78 million people will have dementia by 2030, and 139 million by 2050. Dementia has a significant impact on the individual, the family and the community, in terms of suffering and financial costs, apart from loss. There is no cure, although newer forms of medication are argued to delay deterioration.^1^ There is a genetic predisposition, but genes related to Alzheimer's disease as a causal pathway are distinct from genes that influence modifiable risk factors.^2^ Emerging evidence shows it may be more common in ethnic minorities, and poverty and socioeconomic status are relevant.^3^ An association between dementia and depressive symptoms is also receiving much needed attention by researchers as a potential marker for early intervention.^4^ We have much to do as a research community and as practitioners in order to support people through the process of diagnosis and personalised care, tackle stigma and continue the search for more effective preventive interventions.

**Kamaldeep Bhui**, Professor of Psychiatry, University of Oxford and Editor-in-Chief *BJPsych*

## Background

At the age of 77, having outlived both of my parents, I have recently received a definitive but not unexpected diagnosis of Alzheimer's disease. This followed a computed tomography (CT) brain scan and a thoughtful and detailed assessment by Dr Harwood and his team at the Croydon Memory Clinic. As a psychiatrist with an interest in phenomenology, I thought readers of *the Journal* might be interested in a personal account of the experience of dementia. In addition, given the community and professional response to dementia, I also hope this might make a small contribution to the process of de-stigmatisation.

## First signs of cognitive changes

Some medical problems preceded the development of my cognitive difficulties. I have had moderate hypertension for over 20 years, treated with antihypertensive medication, and probably not quite well enough controlled. I think I first noticed cognitive difficulties after episodes of paroxysmal atrial fibrillation. These were treated in April 2016 and February 2017 under general anaesthesia, and after each treatment I noticed my ability to remember things was, and remained, appreciably reduced. I also noticed that I was having more difficulty in delivering lectures, sometimes failing to remember and to express succinctly the implication of the point I was trying to make. These changes were significant enough for me to discuss the matter with my UCL psychiatric colleague Professor Gill Livingston in January 2018. At that time, she agreed I did have mild cognitive impairment.

In November 2019, as a Biobank participant, I had a CT scan while performing cognitive tasks to the point of failure, and felt there had been a further diminution in my cognitive capacity. I was asked to undertake similar tasks online some weeks before writing this, and once more found them difficult, indeed appreciably more difficult.

## Current situation

Over the past few years my memory has worsened considerably. I have to work harder to remember things that I want to remember, and even then it quite often does not work. Indeed, if I do not concentrate hard, things are occasionally wiped in seconds. I have trouble remembering the names of quite close acquaintances, particularly if I have not seen them for 2 or 3 months. The usual business of forgetting when you cross a threshold what it was you set out to do has become more prominent. I often forget which cupboard to put something in, and unwisely try to compensate by thinking where might be a reasonable option. This is rarely useful.

I have an increased difficulty in dating and ordering events in the medium-term past, although I can often recall a lot of detail in response to prompting. I easily forget future dates, for instance when we are due to take a trip. I sometimes do not register what I am told about events (even significant ones) experienced by other people, which can be socially embarrassing. It has become considerably harder to watch dramas on television because I can lose the plot quite easily. I also have trouble remembering the faces of the characters (an exaggeration of a lifelong propensity).

This is partly an attentional problem: my attention does stray if it is not riveted, and I have to work quite hard to keep focused. Part of the problem is motivational – I sometimes cannot be bothered to deploy the increased energy that I need in order to focus. I sometimes do not finish books even though I initially found them interesting.

Some types of immediate cognitive performance are relatively unimpaired – I can still do the difficult sudokus the hard way quickly and (usually) correctly. I can do things like serial 17s without difficulty (although the fact I even think this is a good idea is indicative).

I have other sorts of cognitive difficulty. I occasionally have a problem in remembering the correct choices when travelling a well-known route. This is a contrast to how I was. I can just about learn a new route following several repetitions. I am still driving, and feel reasonably competent to do so, but keep this under continuous review.

## Déjà vu

Given that Alzheimer's disease has been linked to atrophic changes in the temporal lobe,^5^ I found one of my symptoms of particular interest: I have always been prone to occasional experiences of déjà vu, but these have increased considerably in tandem with my cognitive difficulties.

## Academic work

I find intellectual effort more difficult, although there is some specificity to this. I find it too difficult to do the heavy lifting of formulating and structuring a scientific paper, which I have now given up entirely. However, I do still get a kick out of contributing by commenting on papers written by colleagues. I do not have any trouble finding the words. However, I do have difficulty remembering what exactly is in a paper once it has been out for a few months. Likewise, I now decline invitations to review papers. When I have reviewed a paper, I find it hard to remember the details of my criticisms (I have occasionally discovered, on looking at a paper, that I had already sent in my comments).

I found that I needed to rehearse in detail talks that I had previously presented routinely on teaching courses. As a result, I have now completely stopped presenting papers and lecturing: even after considerable effort and practice I could not rely on getting the sequencing right.

I also have to work a lot harder than I might once have done to learn Italian from Duolingo.

## Impact on personal life

There are clear emotional components to this – obviously I do not like the idea of being unreliable, or of being an inevitable burden to my family, particularly my wife Elizabeth. I do not like feeling stupid. I also notice that I am more emotionally labile – I sometimes feel near to tears when I hear something touching, in a way that is new. I am still prone to disbelieve people who correct me, and sometimes become irritable or even angry, despite being aware of my difficulties (I am working on this!). My self-esteem has always been on the high side, and interestingly appears to remain. This probably contributes to my underestimating the degree of help I need: Elizabeth now takes on all the routine organisational aspects of our lives.

Finally, while I have always had a degree of physical clumsiness, this too has noticeably increased.

I have been on donepezil for the past 6 months: although in controlled trials this reduces the progression of dementia,^1^ there is, of course, no way of knowing its effect in an individual. I have noticed neither treatment effects nor side-effects.

## A recommendation

Finally, I have discovered that by being open about my diagnosis I have received enormous support and affection from colleagues and friends. I recommend this approach!
